# Clinical implementation of PLANET® Dose for dosimetric assessment after [^177^Lu]Lu-DOTA-TATE: comparison with Dosimetry Toolkit® and OLINDA/EXM® V1.0

**DOI:** 10.1186/s13550-020-00737-8

**Published:** 2021-01-04

**Authors:** Lore Santoro, L. Pitalot, D. Trauchessec, E. Mora-Ramirez, P. O. Kotzki, M. Bardiès, E. Deshayes

**Affiliations:** 1 Nuclear Medicine Department, Montpellier Cancer Institute (ICM), Univ. Montpellier, 208 Avenue des Apothicaires, 34298 Montpellier Cedex 5, France; 2 Centre de Recherche en Cancérologie de Toulouse, Toulouse, France; 3 INSERM, UMR 1037, Toulouse III Paul Sabatier University, Toulouse, France; 4 University of Costa Rica, Physics School, CICANUM, San José, Costa Rica; 5 Montpellier Cancer Research Institute, UMR 1194, Univ. Montpellier, Montpellier, France

**Keywords:** Dosimetry workstation, Peptide receptor radionuclide therapy, [^177^Lu]Lu-DOTA-TATE, 3D calibration factor, MIRD, Voxel-based dosimetry

## Abstract

**Background:**

The aim of this study was to compare a commercial dosimetry workstation (PLANET® Dose) and the dosimetry approach (GE Dosimetry Toolkit® and OLINDA/EXM® V1.0) currently used in our department for quantification of the absorbed dose (AD) to organs at risk after peptide receptor radionuclide therapy with [^177^Lu]Lu-DOTA-TATE.

**Methods:**

An evaluation on phantom was performed to determine the SPECT calibration factor variations over time and to compare the Time Integrated Activity Coefficients (TIACs) obtained with the two approaches. Then, dosimetry was carried out with the two tools in 21 patients with neuroendocrine tumours after the first and second injection of 7.2 ± 0.2 GBq of [^177^Lu]Lu-DOTA-TATE (40 dosimetry analyses with each software). SPECT/CT images were acquired at 4 h, 24 h, 72 h and 192 h post-injection and were reconstructed using the Xeleris software (General Electric). The liver, spleen and kidneys masses and TIACs were determined using Dosimetry Toolkit® (DTK) and PLANET® Dose. The ADs were calculated using OLINDA/EXM® V1.0 and the Local Deposition Method (LDM) or Dose voxel-Kernel convolution (DK) on PLANET® Dose.

**Results:**

With the phantom, the 3D calibration factors showed a slight variation (0.8% and 3.3%) over time, and TIACs of 225.19 h and 217.52 h were obtained with DTK and PLANET® Dose, respectively. In patients, the root mean square deviation value was 8.9% for the organ masses, 8.1% for the TIACs, and 9.1% and 7.8% for the ADs calculated with LDM and DK, respectively. The Lin’s concordance correlation coefficient was 0.99 and the Bland–Altman plot analysis estimated that the AD value difference between methods ranged from − 0.75 to 0.49 Gy, from − 0.20 to 0.64 Gy, and from − 0.43 to 1.03 Gy for 95% of the 40 liver, kidneys and spleen dosimetry analyses. The dosimetry method had a minor influence on AD differences compared with the image registration and organ segmentation steps.

**Conclusions:**

The ADs to organs at risk obtained with the new workstation PLANET® Dose are concordant with those calculated with the currently used software and in agreement with the literature. These results validate the use of PLANET® Dose in clinical routine for patient dosimetry after targeted radiotherapy with [^177^Lu]Lu-DOTA-TATE.

## Background

Dosimetry applications are expanding and the number of nuclear medicine departments performing patient dosimetry is growing, especially for patients with neuroendocrine tumours receiving peptide receptor radionuclide therapy [1]. This is possible thanks to the multidisciplinary collaboration between nuclear medicine physicians, medical physicists, and nuclear medicine technologists [2].

In the clinic, many medical teams have developed their own methodology using the tools available in their department and according to their own organizational possibilities [3–5]. This has led to the local implementation of in-house developed dosimetry software and programs [6–10]. However, the legislation on Medical Devices restricts their use to clinical trials. For routine clinical use, a software package must have received the CE mark. Therefore, many medical teams are now acquiring commercial packages, because obtaining the CE label is usually beyond the missions/capabilities of academic structures and due to the increasing availability of commercial software tools. In a previous article we evaluated some commercial packages already on the market [11]. An updated table (Table 1) is presented below, but the field is rapidly and constantly evolving. Only CE-marked software tools are presented (therefore, STRATOS from the Philips research station Imalytics is not included), and some features that are still under development may not have been approved yet.

**Table 1:** List of commercial packages with CE marking

Name	Manufacturer	Image format	VOI/voxel dosimetry	Absorbed dose calculation	References
Dosimetry Toolkit®	GE Healthcare, Waukesha, WI, USA	SPECT/CT Hybrid Planar	VOI (volume of interest)	OLINDA/EXM® V1/V2	https://www.gehealthcare.com/products/molecular-imaging/nuclear-medicine/xeleris-4-dr Stabin et al. [14] Kupitz et al. [15]
PLANET® Dose	DOSIsoft, Cachan, France	SPECT/CT Hybrid	Voxel-based absorbed dose rates calculation with integration over the VOI	Local energy deposition Convolution +/− density correction	https://www.dosisoft.com/products/planet-dose/ Huizing et al. [16]
MIM SurePlan™ MRT	MIM Software Inc., Cleveland, OH, USA	SPECT/CT Hybrid	Voxel-based	Convolution	https://www.mimsoftware.com/mim_sureplan_mrt Maughan et al. [17]
Organ Dosimetry™ Voxel Dosimetry™	Hermes Medical Solutions, Stockholm, Sweden	SPECT/CT Hybrid Planar	VOI/Voxel-based	OLINDA/EXM® V2 Monte Carlo method	https://www.hermesmedical.com/dosimetry/ Hippeläinen et al. [18]
QDOSE®	ABX-CRO, Dresden, Germany	SPECT/CT Hybrid Planar	VOI	IDAC-Dose 2.1 Convolution	https://www.quantitativedose.com Barna et al. [19]

GE, general electric; VOI, volume of interest; SPECT/CT, single photon emission computed tomography/computed tomography; IDAC, internal dose assessment by computer

In our department, dosimetry is integrated in the clinical routine for patients with neuroendocrine tumours treated by peptide receptor radionuclide therapy with [^177^Lu]Lu-DOTA-TATE. Up to now, dosimetry analyses of organs at risk (OAR) have been performed using the combination of Dosimetry Toolkit® (GE Healthcare, Milwaukee, USA) and OLINDA/EXM® V1.0 to calculate the Time Integrated Activity Coefficient (TIAC) and organ-level absorbed dose (AD), respectively [12]. Recently, our department acquired PLANET® Dose (DOSIsoft SA, Cachan, France), a new CE-marked commercial dosimetry workstation. The initial motivation for changing was to use a vendor-neutral solution to support multicentric trials and to put in place a central dosimetry system. However, before its clinical routine implementation, we wanted to compare this new dosimetric package with our internal reference. Our validation plan involved comparing the results obtained on phantom (calibration factor, TIAC) and in patients (mean AD to OARs, TIACs and organ volumes), using similar parameters in terms of segmentation, registration, and time activity curve fitting. This allowed assessing the consistency between the dosimetric results obtained using Dosimetry Toolkit® and OLINDA/EXM® V1.0 (our reference) and with PLANET® Dose.

## Material and methods

### Dosimetry software packages

The characteristics of the two dosimetry software packages used in this study are presented in Table 2.

**Table 2:** General characteristics for each step of the dosimetry workflow in the currently used dosimetry “Dosimetry Toolkit + OLINDA” software (the reference in this study), and the new PLANET® Dose package

	Dosimetry Toolkit® + OLINDA/EXM® V1.0	PLANET® Dose
Calibration procedure	GE recommendations: planar acquisitions Calibration factor: counts.s^−1^.MBq^−1^	No manufacturer’s recommendations Calibration factor: Bq.Count^−1^
Clinical imaging expected	Whole body scans, SPECT/CT or hybrid 5 time points maximum Only from GE devices	SPECT/CT or hybrid Unlimited time points Import in DICOM format from all devices
Reconstruction	« Preparation for Dosimetry Toolkit» application	Non applicable
Registration	« Preparation for Dosimetry Toolkit» application Automatic rigid registration between CT images (Full FOV)	Rigid or elastic registration Using CT data
Segmentation and propagation	Manual, semi-automatic or automatic segmentation using the first images (NM or CT) Rigid propagation (constant volumes) Segmentation adjusted by translation or rotation on others CT images	Manual, semi-automatic or automatic segmentation using the first images (NM or CT) Rigid propagation (constant volumes) No adjustment on others CT images
TIA fitting	Mono-exponential exclusively	Mono-exponential, Bi-exponential, Xexp, Trapezoidal, Tri-exponential, …
Absorbed dose calculation	Not avalaible on Dosimetry Toolkit® TIAC exported to OLINDA/EXM® V1.0	Local Deposition Method (LDM) Dose Kernel (DK) approach with / without density correction
Import/export	Not applicable	DICOM format (DICOM-RT-Structure and RT-Dose)

GE, general electric; FOV, field of view; NM, nuclear medicine; CT, computed tomography; TIA, Time tntegrated activity; TIAC, time integrated activity coefficient; DICOM-RT, digital imaging and communications in medicine-radiotherapy

Dosimetry Toolkit® is an application of the Xeleris® software (GE Healthcare, Milwaukee, USA) [13, 15]. GE Healthcare recommends to use a procedure based on planar acquisition to determine the calibration factor (CF; in counts.s^−1^.MBq^−1^). For clinical dosimetry, different scenarios are available: whole-body, hybrid, or multi-SPECT/CT image acquisition. It includes two steps. The first, “Preparation for Dosimetry Toolkit”, is used for the reconstruction of SPECT/CT raw data and registration of the CT or planar whole body images. The second, “Dosimetry Toolkit”, is used to segment the different organs, create the time activity curves fitted by a mono-exponential function, and calculate the TIAC for each of them. These TIACs are then uploaded in OLINDA/EXM® V1.0 [14] to calculate the organ mass-adjusted ADs. At the time of the study, only OLINDA V1 was available. The future version of Dosimetry Toolkit should propose OLINDA V2, but any model-based dosimetry software that includes S values (IDAC-Dose for example) could be used [20].

PLANET® Dose is a treatment planning system from DOSIsoft. The calibration procedure is left to the user’s discretion and a CF (in Bq.count^−1^) is required. This dosimetry package does not reconstruct SPECT/CT data, but accepts reconstructed data in DICOM format from all devices. It provides multi-time point registration (using rigid and elastic algorithms), organ segmentation (manual and automatic), and TIAC calculation with a wide choice of interpolation methods of the time-activity curve (linear, trapezoidal, mono-, X-, bi-, tri-exponential…). The mean AD can be calculated by assuming the local energy deposition (local deposition model—LDM) or by convolution of AD voxel kernels (dose kernels—DK), with or without media density correction [21–23].

For this study, the segmentation, registration and TIAC steps were carried out on PLANET® Dose using parameters similar to those defined in Dosimetry Toolkit® to allow the comparison.

### Dosimetry imaging protocol

All imaging acquisitions were performed with a SPECT/CT Discovery NM/CT 670 (General Electric [GE] Healthcare), including a BrightSpeed 16 CT scanner and a 3/8-inch NaI(Tl) crystal, according to the acquisition protocol previously described [12]. Briefly, nuclear medicine images were acquired using a medium-energy general purpose parallel-hole collimator. A 20% energy window centred on the 208 keV photopeak and a 10% scatter correction window centred on 177 keV were applied. NM acquisitions were performed using a body contour option, rotation of 180° per detector, total of 60 projections and 45 s each. For attenuation correction, CT images were acquired (120 kV, automatic mA regulation with a max at 200 mA, noise index at 6.43, slice thickness of 5 mm, rotation time of 0.8 s, pitch 1.375, 512 × 512 pixels matrix), with standard reconstruction.

The application “Preparation for Dosimetry Toolkit” was used for SPECT/CT image reconstruction for both dosimetry approaches. The Ordered Subset Expectation Maximization iterative reconstruction algorithm was used with 6 iterations and 10 subsets, attenuation, scatter, recovery resolution corrections and a Gaussian post-filter of 0.11 cm [12].

### Phantom study

#### Calibration factor and time integrated activity coefficient

**Fig. 1:**
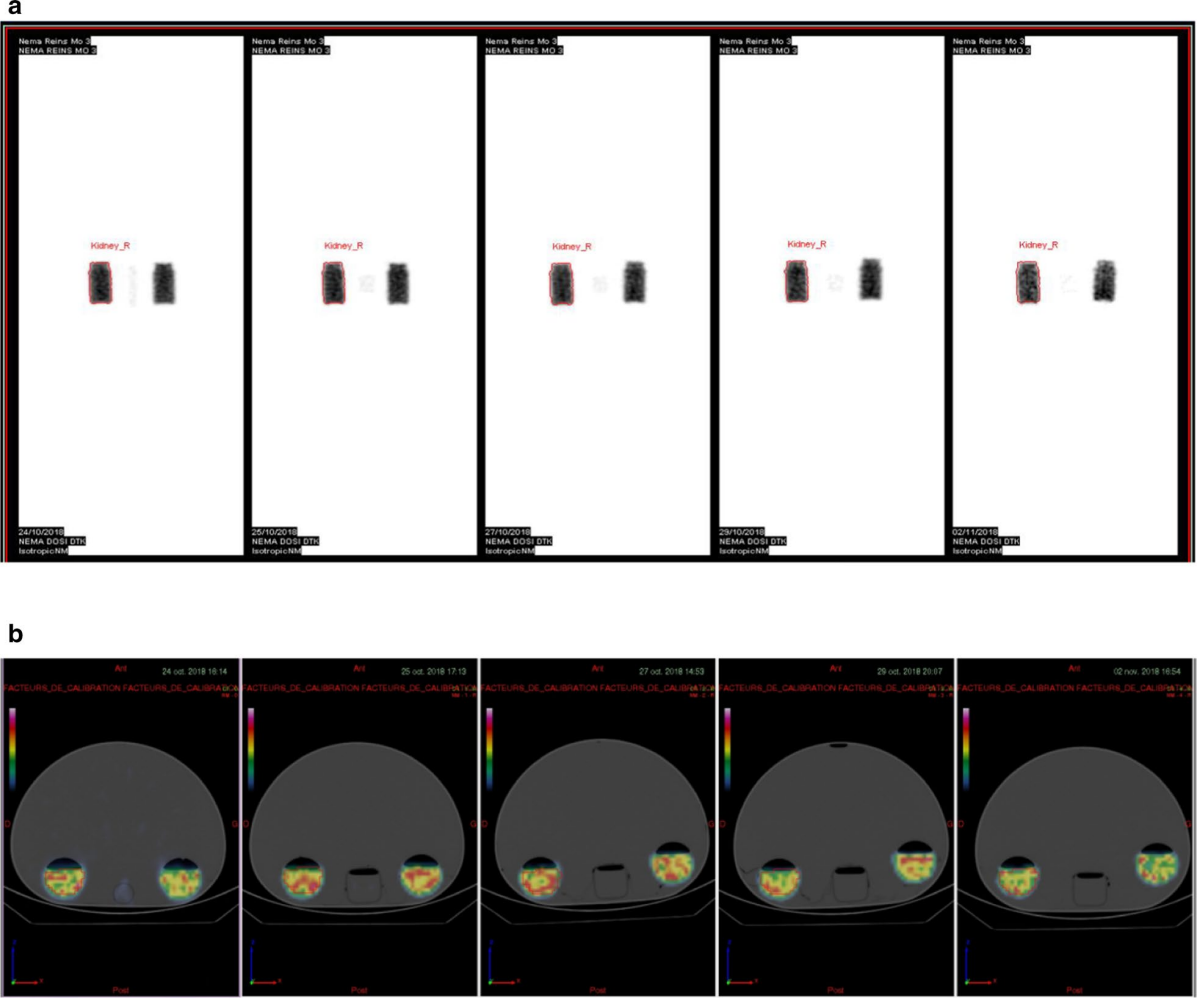
Dosimetry Toolkit® (**a**) and PLANET® Dose interfaces (**b**) after imaging of the NEMA IEC body phantom (Body Phantom NU2-2001/2007) that contains two 250 mL bottles filled with 200 mL of [^177^Lu]Lu-DOTA-TATE at different time points. Only one of the two bottles was segmented

A NEMA IEC body phantom (Body Phantom NU2-2001/2007) that contains two bottles of 250 mL filled with 200 mL of 82.2 ± 4.1 (i.e. maximum activity measurement error of 5%) MBq [^177^Lu]Lu-DOTA-TATE was chosen with the aim of mimicking the size of kidneys. The background was filled with non-radioactive water. SPECT/CT images were acquired at different time points to evaluate CF variations over time. The CF was estimated using one of the two bottles filled with [^177^Lu]Lu-DOTA-TATE (Fig. 1).

##### Dosimetry toolkit

A CT rigid registration based on the full phantom was performed with “Preparation for Dosimetry Toolkit”. Then, using the “Dosimetry Toolkit” application, an isocontour representing a volume of 200 mL was automatically segmented on the first nuclear medicine image and was replicated for the images at 24 h, 72 h, 120 h and 216 h. For each time-point, the segmented volume was kept constant. To determine the CF in counts.s^−1^.MBq^−1^ at each time point, the number of events in the volume was divided by the acquisition time provided by the DICOM data (2700 s) and by the activity. For radioactivity decay correction, a physical half-life of 6.647 days [24] was applied and the activity at each acquisition time point was corrected for the phantom preparation time. The CF was calculated as the mean of the CF values obtained at the different time points. To obtain the time-activity curve fitted by a mono-exponential function and the TIAC (h), information about the radionuclide and the CF were entered in the appropriate interface.

##### PLANET® Dose

For the body phantom, CT image rigid registration based on the volume that tightly enclosed the bottle was performed with PLANET® Dose. Similarly, an isocontour that represented a volume of 200 mL was automatically segmented on the first functional image and strictly transferred to the SPECT/CT images acquired at the other time points using a rigid contour propagation algorithm. The segmented volume was maintained over time. To determine the CF in Bq.count^−1^ at each time point, the number of events in the segmented volume was divided by the activity in Bq, by taking into account the radioactivity decay. The CF was calculated as the mean of the CF values obtained at the different time points.

A mono-exponential fitting function, similar to the Dosimetry Toolkit® approach, was used to calculate the TIA $\widetilde{A}$, and the TIAC $\tau$, as [25]:

$$\ \widetilde{A} = \left[ {\frac{{A_{0} \times T_{{\text{e}}} }}{\ln 2}} \right]\quad {\text{and}}\quad \tau = \left[ {\frac{1}{\ln 2}} \right] \times T_{{\text{e}}} = 230.15 \,{\text{h}}$$

where $A_{0}$ is the initial activity, and $T_{{\text{e}}}$ is the experimental half-life assessed using a mono-exponential fit.

As in that situation, only physical decay is observed (*T* = 6.647 days) [24], it is possible to derive the reference TIAC $\tau_{{{\text{ref}}}}$ as:

$$\tau_{{{\text{ref}}}} = \left[ {\frac{1}{\ln 2}} \right] \times T = 230.15 \,{\text{h}}$$

The reference TIAC $\tau_{{{\text{ref}}}}$ was compared to the experimental $\tau$ values obtained with the two dosimetry platforms.

### Clinical study

#### Patients and treatment

Twenty-one patients (5 women and 16 men; median age 68 years, range 41–82 years) with a neuroendocrine tumour and treated with [^177^Lu]Lu-DOTA-TATE, Lutathera® (Advanced Accelerator Applications, Saint Genis Pouilly, France) were evaluated (Additional file 1: Table S1). The treatment consisted in 7.2 ± 0.2 GBq activity (four infusions in total) injected every 8 weeks. Amino acids (lysine + arginine) were administered concomitantly to ensure renal protection by reducing tubular reabsorption of the radiolabelled peptides. All patients were hospitalized in specialized radioprotection rooms for 24 h after injection. Patients were then discharged and had to come back for the other post-infusion imaging sessions. Dosimetry to liver, kidneys and spleen (i.e. the OARs) was performed after the first and second infusion of [^177^Lu]Lu-DOTA-TATE. Dosimetry data after the first injection were not evaluable in one patient, and another patient died before the second infusion. In total, 40 dosimetry analyses were performed with each dosimetry package.

#### Dosimetry workflow

The dosimetry workflow for the two packages is presented in Table 3. SPECT/CT images were acquired at 4 h, 24 h, 72 h and 192 h after infusion. For some patients, due to health problems, technical issues or calendar reasons, SPECT/CT images were acquired at only three time points after injection. As dosimetry for the first two injections of [^177^Lu]Lu-DOTA-TATE is performed routinely in our department, currently with Dosimetry Toolkit + OLINDA, we carried out a retrospective additional analysis of the already available dosimetric data with PLANET® Dose.

**Table 3:** Dosimetry workflow in patients using the reference dosimetry approach Dosimetry Toolkit + OLINDA and the new workstation PLANET® Dose

	Dosimetry Toolkit® + OLINDA/EXM® V1.0	PLANET® Dose
Calibration procedure	SPECT/CT acquisitions Calibration factor (counts.s^−1^.MBq^−1^)	SPECT/CT acquisitions Calibration factor (Bq.Count^−1^)
Clinical imaging	4 SPECT/CT at 4 h, 24 h, 72 h and 192 h after injection—60 projections of 45 s	
Reconstruction	« Preparation for Dosimetry Toolkit» 6 iterations 10 subsets-AC, SC, RR, Gaussian post filter 0.11 cm	
Registration	« Preparation for Dosimetry Toolkit» application Automatic rigid registration using CT images (Full FOV)	Organ-based rigid registration using CT images
Segmentation and propagation	Manual segmentation using the first CT image Rigid propagation (constant volumes) Segmentation adjusted by translation or rotation for other images	Manual segmentation using the first CT image Rigid propagation (constant volumes) No adjustment for other images
TIA fitting	Mono-exponential	Mono-exponential
Absorbed dose calculation	OLINDA/EXM® V1.0 Patient-adapted organ masses	Local Deposition Method (LDM) Dose Kernel (DK) approach with/without density correction

AC, attenuation correction; SC, scatter correction, RR, recovery resolution; FOV, field of view; CT, computed tomography; TIA, time integrated activity

##### Reference dosimetry method

For both infusions, after the last SPECT/CT image acquisition at 192 h, all SPECT/CT data were loaded on the “Preparation for Dosimetry Toolkit” application. Imaging data were reconstructed and an automatic rigid registration of the CT scans was performed. The results were loaded on the “Dosimetry Toolkit” application. Liver, kidneys and spleen were manually segmented using the CT images collected at 4 h post-injection, and then the segmented contour was rigidly propagated to the 24 h, 72 h, and 192 h images. For each time point, the segmented volume was maintained, but sometimes it was adjusted by translation or rotation. The partial volume effect correction was considered negligible. The administered activity, date and time of administration, radionuclide and CF values (in counts.s^−1^.MBq^−1^) were entered. To obtain the TIAC, the time-activity curves were fitted using a mono-exponential function, the only fitting model available in the “Dosimetry Toolkit” application. Then, the TIAC values were exported to OLINDA/EXM® V1.0 to calculate the ADs to liver, kidneys and spleen (i.e. the OARs). The organ masses included in this software were determined from the organ volume defined on the CT images using “Dosimetry Toolkit” and the biological tissue density (1.06 g.cm^−3^ for liver and spleen; 1.05 g.cm^−3^ for kidneys).

##### PLANET® Dose

The transversal slices reconstructed using the “Preparation for Dosimetry Toolkit” application and the corresponding CT images were uploaded on PLANET® Dose. Automatic and rigid registration was performed iteratively for each organ, based on a volume tightly enclosing the organ of interest. Then, the organs of interest on the first CT image were manually segmented, and the volumes were transferred from the reference CT image to the SPECT/CT images acquired at the other time points by rigid propagation. The volume of each OAR remained constant at all time points. As mentioned above, the partial volume effect correction was considered negligible for the OARs under study.

The administered activity, date and time of administration, radionuclide used, and CF value (in Bq.counts^−1^) were entered. The time-activity curve was fitted using a similar approach as the one used in Dosimetry Toolkit® (i.e*.* mono-exponential function) to provide the TIAC (h) and the TIA (MBq.s). The mean ADs were calculated using the LDM and DK methods, with correction of density.

#### Mean absorbed dose calculation

**Fig. 2:**
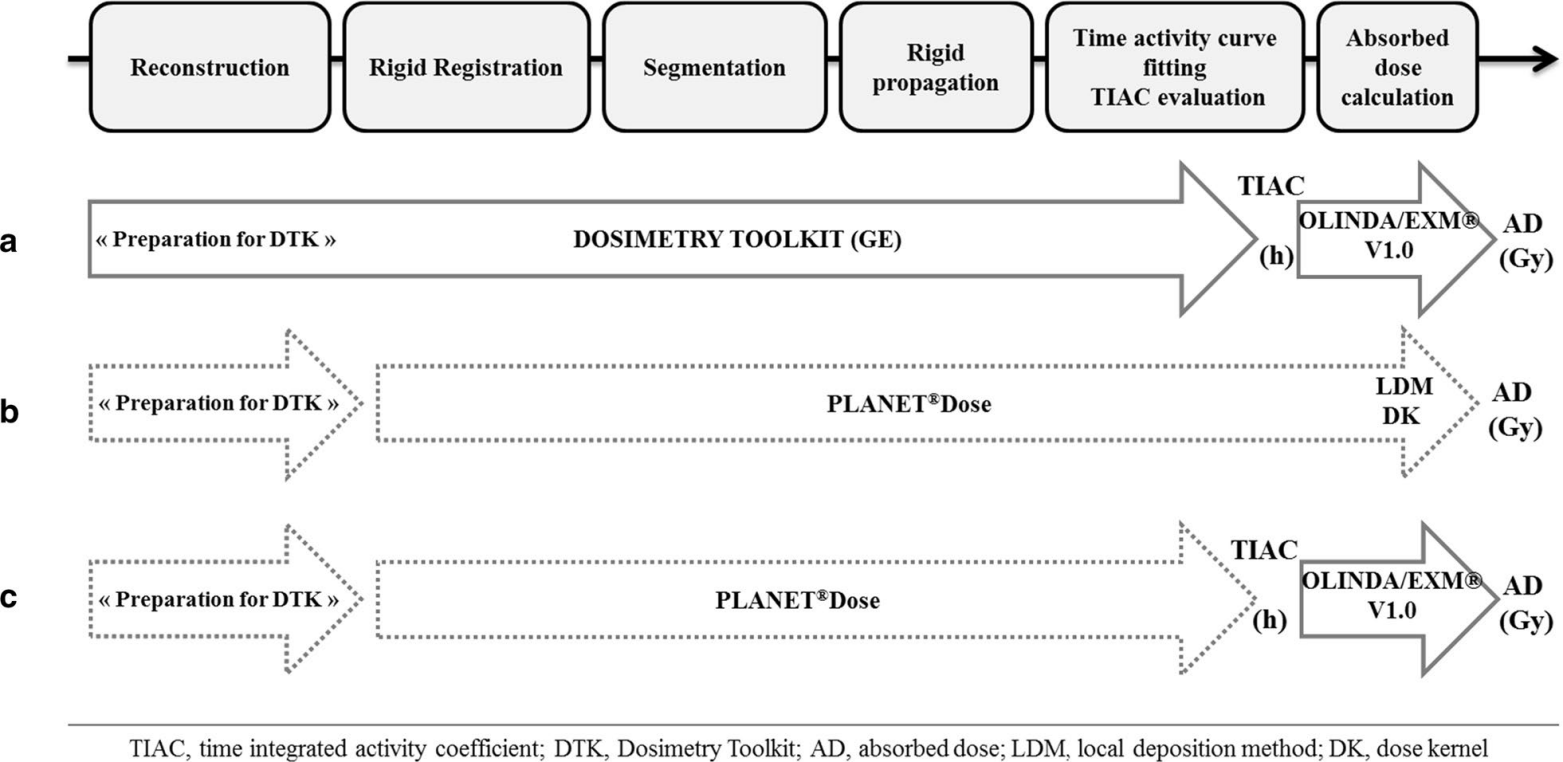
Dosimetry workflow using the reference dosimetry approach Dosimetry Toolkit + OLINDA (**a**) and the PLANET® Dose package with the LDM and DK methods (**b**). The PLANET® Dose + OLINDA approach (**c**) was used to evaluate independently the AD calculation methods

The mean AD was calculated using the first two approaches presented in Fig. 2:

- The “Dosimetry Toolkit” approach used TIAC provided by Dosimetry Toolkit®, entered in OLINDA/EXM® V1.0, to derive the mean ADs to liver, kidneys and spleen (Fig. 2a).
- The PLANET® Dose approach used reconstructed images (Preparation for Dosimetry Toolkit) and full processing (registration, segmentation, TIAC and AD calculation using the LDM and DK methods, with density correction) on PLANET® Dose (Fig. 2b).

A third approach was also used to evaluate independently the previous two AD calculation methods. This approach was similar to the PLANET® Dose method, but after the TIAC step, the ADs were computed with OLINDA/EXM® V1.0 (Fig. 2c) using the same phantom model than in the first approach (adult male or female).

#### Statistical analyses

Besides the relative difference (in %), the root mean-square deviation (RMSD) of organ masses, TIACs and ADs per cycle obtained with PLANET® Dose and Dosimetry Toolkit + OLINDA (taken as reference) was calculated as follows:

$${\text{RMSD}} = \sqrt {\frac{{\mathop \sum \nolimits_{i} \left[ {\frac{{\left( {X_{{{\text{PLANET dose}}\,i}} - X_{{{\text{DTK Olinda }}\,i}} } \right)}}{{X_{{{\text{DTK Olinda }}\,i}} }}} \right]}}{{\text{number of dosimetry analysis}}}}$$

where *X*_(*i*)_ was the organ masses, TIACs or ADs obtained for the dosimetry analysis *i*.

The Lin’s concordance correlation coefficient [26] was used to evaluate the agreement between PLANET® Dose LDM and the reference method (Dosimetry Toolkit + OLINDA). This analysis was performed using the mean values for all patients and organs after the two infusions. Moreover, the absolute differences between the mean AD values obtained with the two approaches were assessed for each organ using the Bland–Altman plot analysis [27]. The 95% limits of agreement, from − 1.96 to + 1.96 SD, were calculated for each organ.

The paired Student’s *t*-test was used to compare the liver, kidneys and spleen ADs calculated with Dosimetry Toolkit + OLINDA and PLANET® Dose LDM (*n* = 40). This analysis was performed using the mean values for all patients and organs after the two infusions.

To evaluate independently the AD calculation methods, the mean AD obtained with PLANET® Dose and with “PLANET® Dose + OLINDA/EXM® V1.0” (taken as reference) were compared. A bland–Altman analysis and a correlation evaluation were performed.

## Results

### Phantom-based study

**Fig. 3:**
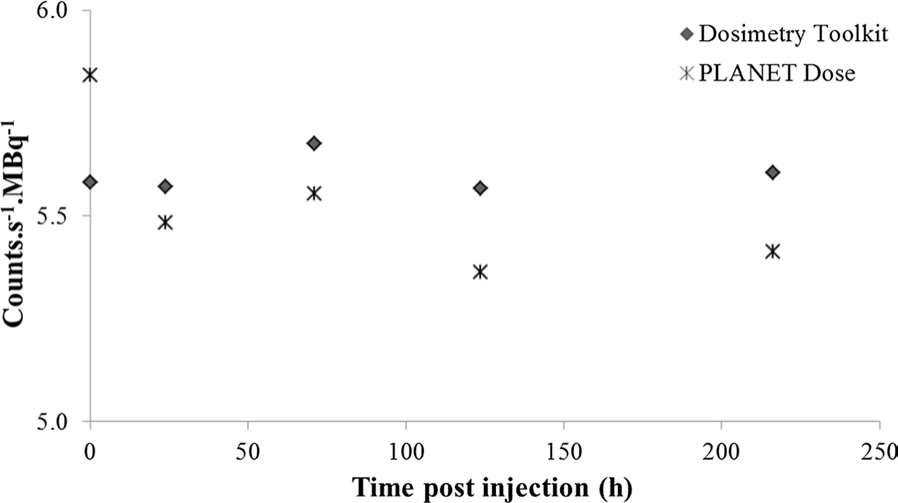
Variation of the SPECT CF values over time after [^177^Lu]Lu-DOTA-TATE injection obtained with Dosimetry Toolkit® and PLANET® Dose

SPECT/CT CF values of 5.60 ± 0.04 counts.s^−1^.MBq^−1^ and 5.53 ± 0.19 counts.s^−1^.MBq^−1^ were obtained with Dosimetry Toolkit® and PLANET® Dose, respectively. These values did not vary significantly over time (0.8% and 3.3% of variation, respectively) (Fig. 3). For PLANET® Dose, the CF value was converted to 67 ± 2.2 Bq.counts^−1^ to be uploaded on the software.

These CF values were then used in the clinical study to calculate the time activity curves and TIACs for liver, kidneys, and spleen.

TIACs of 217.52 h and 225.19 h were obtained with Dosimetry Toolkit and PLANET® Dose, respectively. These values showed a deviation of − 5.5% and − 2.2% relative to the reference residence time of 230.15 h.

### Clinical results

#### Package comparison

The mean organ masses, TIACs and ADs for liver, kidneys and spleen (i.e. OARs) obtained using each dosimetry package are summarized in Table 4a. The relative differences of the organ mass values and TIACs between Dosimetry Toolkit + OLINDA/EXM® V1.0 (the reference) and PLANET® Dose are presented in Table 4b. These results highlighted RMSD values lower than 10% for organ mass values and TIACs, but for the spleen TIAC (RMSD = 10.4%).

**Table 4:** (a) Mean and standard deviation (*n* = 40 dosimetry analyses) of OAR organ masses, TIACs and ADs calculated using Dosimetry Toolkit + OLINDA/EXM® V1.0 and PLANET® Dose with LDM and DK and density correction. (b) Relative differences (%) of the organ mass values and TIACs between PLANET® Dose and Dosimetry Toolkit + OLINDA, DTK, Dosimetry Toolkit

(a)							
ORGANS	Mass (g)		TIAC (h)		Absorbed dose (Gy)		
	DTK + OLINDA/EXM® V1.0	PLANET®Dose	DTK + OLINDA/EXM® V1.0	PLANET® Dose	DTK + OLINDA/EXM® V1.0	PLANET®Dose	
						LDM	DK
Liver	2141.6 ± 1213.3	2191.7 ± 1205	14.9 ± 24.1	15.1 ± 24.2	3.40 ± 3.9	3.27 ± 3.7	3.21 ± 3.6
Kidneys	478.3 ± 111.4	461.5 ± 108.3	2.2 ± 0.7	2.4 ± 0.8	3.01 ± 0.9	3.23 ± 0.9	3.16 ± 0.9
Spleen	290.4 ± 181.3	281.7 ± 178.5	2.0 ± 1.5	2.0 ± 1.4	4.15 ± 1	4.45 ± 1.2	4.36 ± 1.2

(b)						
Relative difference (%)/DTK+OLINDA						
	Mass			TIAC		
	Liver	Kidneys	Spleen	Liver	Kidneys	Spleen
Mean	2.9	−3.4	−2.4	5.7	6.4	4.9
Min	−4.0	−18.8	−23.4	−6.9	−9.1	−18.9
Max	19.7	16.4	17.3	17.3	25.7	23.5
RMSD	5.2	7.2	8.9	8.1	9.4	10.4

**Fig. 4:**
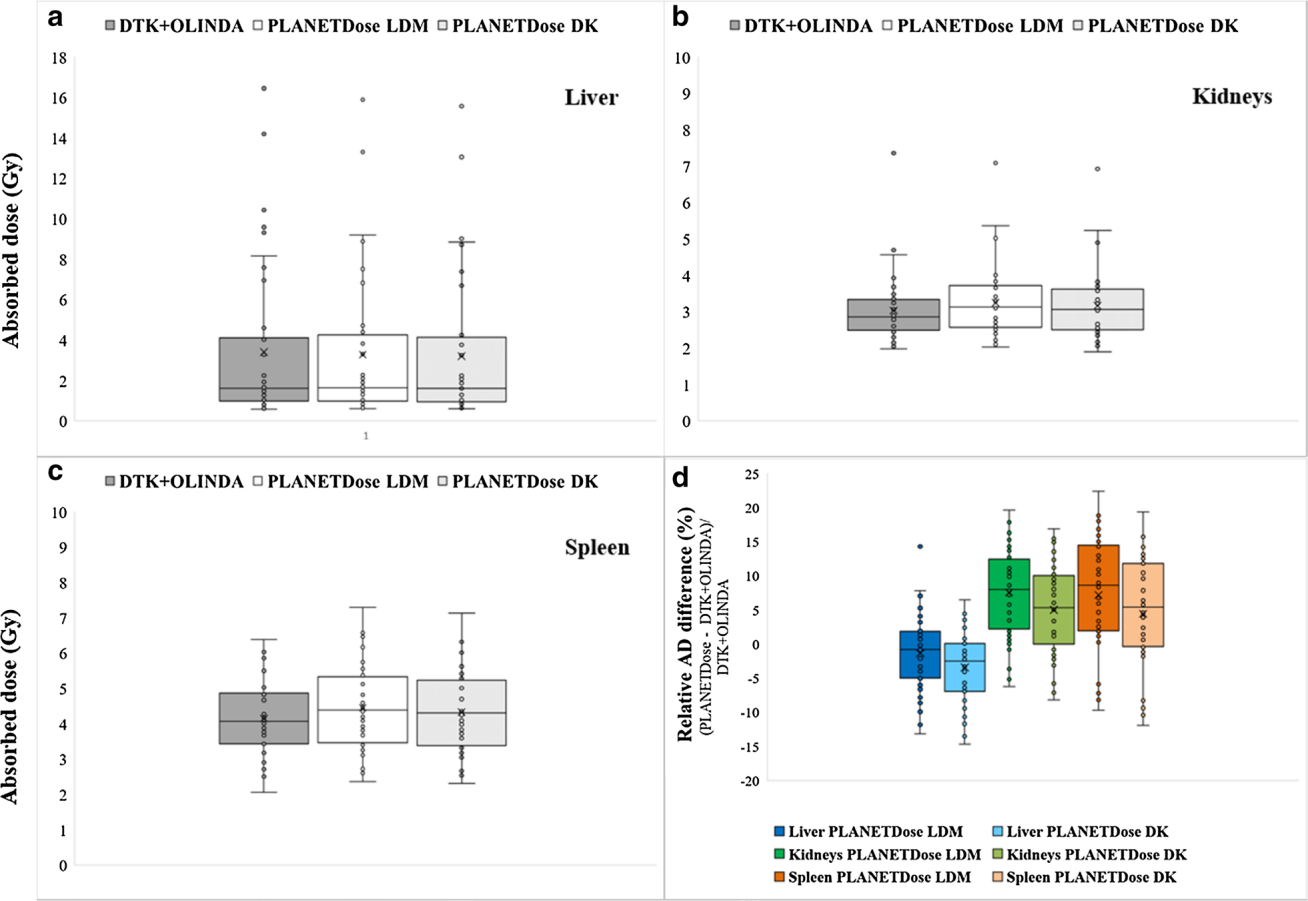
Box-and-whisker plots showing the ADs to liver (**a**), kidneys (**b**) and spleen (**c**) calculated using Dosimetry Toolkit + OLINDA and the PLANET® Dose packages, as well as the relative differences of AD values between PLANET® Dose and Dosimetry Toolkit + OLINDA (**d**). DTK, Dosimetry Toolkit

The comparison of the OAR AD values obtained with Dosimetry Toolkit + OLINDA and with PLANET® Dose, LDM and DK, are presented in Table 5a–c and Fig. 4. Again, the mean difference and RMSD values were lower than 10% for all organs, except for spleen (RMSD = 10.9%). For kidneys and spleen, the AD values obtained with PLANET® Dose were slightly, but significantly higher (*p* < 0.05).

**Table 5:** Comparison of the AD values to each OAR: liver (a), kidneys (b) and spleen (c) calculated with Dosimetry Toolkit + OLINDA and PLANET® Dose with density correction. DTK, Dosimetry Toolkit

(a)					
Dosimetry analyses	Absorbed dose to liver (Gy)				
	DTK + OLINDA/EXM® V1.0	PLANET®Dose LDM	PLANET®Dose DK	Relative difference (%)/DTK + OLINDA	
				PLANET®Dose LDM	PLANET®Dose DK
1	1.04	1.04	1.02	− 0.1	− 1.9
2	1.59	1.64	1.61	3.2	1.2
3	0.92	0.86	0.85	− 6.7	− 8.3
4	16.44	15.89	15.56	− 3.4	− 5.4
5	14.19	13.31	13.06	− 6.2	− 8.0
6	3.27	3.30	3.24	0.9	− 1.0
7	4.59	4.40	4.32	− 4.1	− 5.9
8	0.57	0.61	0.60	7.0	4.4
9	2.05	2.04	2.00	− 0.3	− 2.2
10	1.26	1.09	1.07	− 13.2	− 14.7
11	1.12	1.11	1.10	− 0.9	− 2.2
12	2.40	2.28	2.23	− 5.0	− 7.0
13	4.02	3.83	3.76	− 4.7	− 6.4
14	0.91	0.93	0.92	2.1	0.7
15	0.79	0.80	0.78	1.3	− 1.0
16	9.58	9.00	8.83	− 6.0	− 7.8
17	9.32	8.88	8.72	− 4.7	− 6.4
18	1.47	1.49	1.44	1.5	− 1.9
19	1.92	1.89	1.86	− 1.8	− 3.3
20	0.94	0.86	0.84	− 8.7	− 10.7
21	1.13	1.18	1.16	4.1	2.4
22	1.65	1.68	1.65	1.8	0.1
23	0.88	0.93	0.91	5.7	3.5
24	6.95	6.89	6.75	− 0.9	− 2.8
25	2.09	2.10	2.06	0.7	− 1.1
26	2.34	2.29	2.25	− 2.1	− 3.8
27	7.57	6.82	6.69	− 9.9	− 11.7
28	0.62	0.65	0.63	5.2	2.7
29	1.61	1.64	1.61	1.9	0.1
30	1.14	1.14	1.12	− 0.4	− 2.3
31	1.39	1.31	1.29	− 5.6	− 7.0
32	2.24	2.19	2.15	− 2.1	− 4.1
33	4.13	4.72	4.25	14.3	2.9
34	0.78	0.84	0.83	7.7	6.5
35	0.81	0.81	0.80	0.0	− 1.6
36	10.43	9.19	9.02	− 11.9	− 13.5
37	8.15	7.51	7.38	− 7.9	− 9.5
38	1.42	1.36	1.34	− 4.1	− 5.7
39	1.57	1.49	1.46	− 5.1	− 6.8
40	0.92	0.94	0.93	2.0	0.5
Mean	3.40	3.27	3.20	− 1.4	− 3.5
SD	3.89	3.65	3.58	5.5	4.9
Min	0.57	0.61	0.60	− 13.2	− 14.7
Max	16.44	15.89	15.56	14.3	6.5
RMSD				5.6	6.0

(b)					
Dosimetry analyses	Absorbed dose to kidneys				
	DTK + OLINDA/EXM® V1.0	PLANET®Dose LDM	PLANET®Dose DK	Relative difference (%)/DTK + OLINDA	
				PLANET®Dose LDM	PLANET®Dose DK
1	2.78	3.14	3.07	12.8	10.3
2	2.79	3.10	3.03	11.1	8.5
3	3.26	3.41	3.33	4.6	2.1
4	3.68	3.45	3.38	− 6.3	− 8.2
5	2.05	2.03	1.91	− 0.7	− 6.9
6	3.06	3.66	3.57	19.6	16.8
7	3.56	3.84	3.75	7.7	5.1
8	3.48	3.83	3.70	10.0	6.2
9	2.53	2.40	2.35	− 5.2	− 7.2
10	3.07	3.29	3.22	7.2	5.0
11	2.10	2.40	2.34	14.3	11.5
12	2.51	2.61	2.56	3.9	1.8
13	3.24	3.35	3.28	3.4	1.1
14	2.44	2.72	2.66	11.5	8.8
15	2.20	2.50	2.45	13.6	11.1
16	1.98	2.10	2.06	5.8	3.8
17	2.96	3.49	3.41	18.0	15.4
18	2.90	3.13	3.05	8.1	5.4
19	4.69	5.37	5.24	14.5	11.7
20	2.48	2.46	2.40	− 0.8	− 3.2
21	3.31	3.73	3.65	12.7	10.2
22	2.85	3.15	3.12	10.7	9.5
23	3.93	4.00	3.91	1.8	− 0.5
24	2.60	2.60	2.54	0.0	− 2.3
25	3.54	3.91	3.82	10.5	8.0
26	3.31	3.50	3.42	5.7	3.3
27	7.36	7.09	6.93	− 3.7	− 5.8
28	3.35	3.86	3.76	15.2	12.3
29	2.51	2.55	2.49	1.6	− 0.8
30	3.32	3.34	3.27	0.6	− 1.6
31	2.62	2.82	2.75	7.5	5.0
32	2.31	2.55	2.49	10.5	8.0
33	3.37	3.92	3.83	16.3	13.5
34	2.53	2.68	2.62	5.9	3.5
35	2.30	2.71	2.64	17.8	14.9
36	2.15	2.22	2.17	3.4	1.2
37	2.08	2.10	2.06	1.2	− 0.8
38	2.87	3.11	3.04	8.5	6.0
39	4.57	5.02	4.90	9.8	7.2
40	2.52	2.80	2.73	11.1	4.6
Mean	3.03	3.25	3.17	7.5	4.9
SD	0.95	0.97	0.95	6.5	6.4
Min	1.98	2.03	1.91	− 6.3	− 8.2
Max	7.36	7.09	6.93	19.6	16.8
RMSD				9.9	8.0

(c)					
Dosimetry analyses	Absorbed dose to spleen				
	DTK + OLINDA/EXM® V1.0	PLANET®Dose LDM	PLANET®Dose DK	Relative difference (%)/DTK + OLINDA	
				PLANET®Dose LDM	PLANET®Dose DK
1	3.86	4.12	4.03	6.8	4.5
2	4.67	5.41	5.28	15.8	13.1
3	3.83	4.40	4.31	15.0	12.5
4	4.01	3.68	3.59	− 8.2	− 10.4
5	4.35	3.93	3.83	− 9.7	− 12.0
6	2.98	3.30	3.23	10.9	8.3
7	5.03	5.37	5.28	6.7	5.0
8	2.07	2.36	2.31	14.3	11.7
9	3.67	3.40	3.32	− 7.2	− 9.4
10	4.87	4.59	4.37	− 5.7	− 10.2
11	3.69	3.83	3.74	3.8	1.4
12	4.27	4.35	4.25	1.8	− 0.5
13	4.17	4.47	4.37	7.2	4.7
14	3.22	3.30	3.22	2.5	0.0
15	2.91	3.33	3.26	14.6	12.0
16	2.94	3.11	3.05	5.9	3.8
17	2.57	2.60	2.54	1.1	− 1.2
18	4.75	5.19	5.07	9.3	6.8
19	5.50	6.17	6.02	12.2	9.5
20	3.77	3.94	3.86	4.6	2.4
21	4.83	5.64	5.51	16.8	14.2
22	3.88	4.38	4.35	12.9	12.1
23	4.91	5.55	5.42	13.0	10.4
24	4.05	3.81	3.71	− 5.9	− 8.3
25	6.38	7.30	7.13	14.4	11.8
26	4.18	4.53	4.42	8.3	5.7
27	5.57	5.12	5.02	− 8.1	− 9.9
28	2.50	2.72	2.66	8.8	6.4
29	3.43	4.20	4.10	22.4	19.3
30	4.75	4.82	4.70	1.5	− 1.0
31	5.52	6.51	5.67	18.0	2.8
32	4.09	4.46	4.36	9.0	6.6
33	5.02	5.75	5.63	14.5	12.0
34	2.71	2.80	2.73	3.3	0.6
35	3.44	4.09	3.98	18.8	15.7
36	3.81	4.41	4.31	15.8	13.3
37	3.18	3.25	3.18	2.2	− 0.1
38	5.86	6.46	6.32	10.2	7.8
39	6.03	6.57	6.40	9.0	6.1
40	4.84	4.85	4.75	0.2	− 1.9
Mean	4.15	4.45	4.33	7.2	4.4
SD	1.03	1.19	1.14	8.3	8.0
Min	2.07	2.36	2.31	− 9.7	− 12.0
Max	6.38	7.30	7.13	22.4	19.3
RMSD				10.9	9.1

The mean difference between the AD values calculated with LDM and DK (PLANET® Dose) was 2.2%. The values obtained with the LDM method were always higher than those obtained with the DK method (Fig. 4d). Moreover, whatever the software used, liver presented the highest AD variability, with the highest values reaching 16 Gy (Fig. 4a).

#### Concordance between packages

**Fig. 5:**
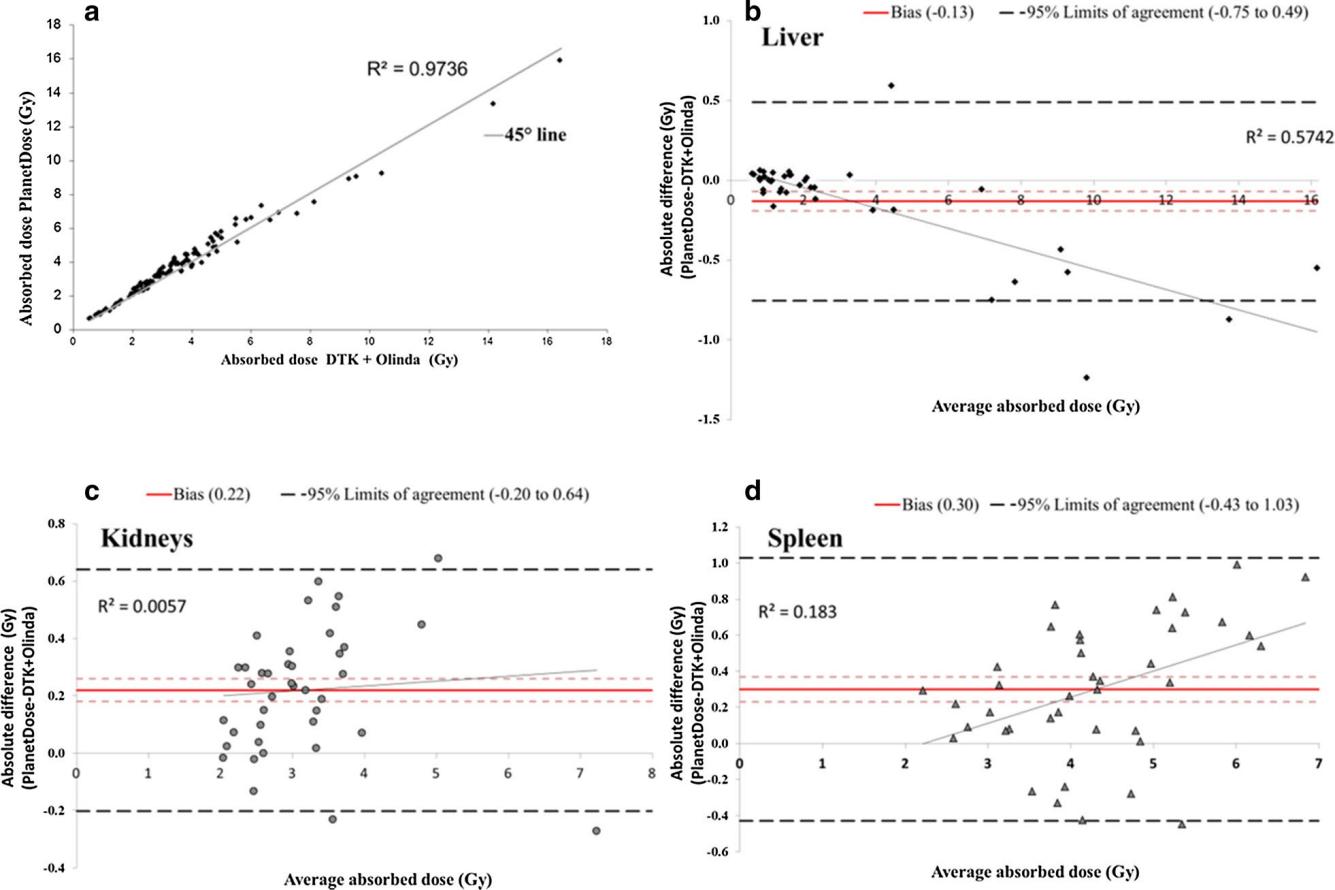
Dispersion around the 45° line of the AD pairs obtained with Dosimetry Toolkit + OLINDA and PLANET® Dose LDM with density correction for all organs combined (**a**). Bland–Altman plots of the ADs to liver (**b**), kidneys (**c**) and spleen (**d**), calculated with Dosimetry Toolkit + OLINDA and PLANET® Dose LDM with density correction. DTK, Dosimetry Toolkit

For the 40 dosimetry evaluations, the estimated Lin’s concordance correlation coefficient was 0.99 (95% CI 0.99; 0.99; *R*^2^ = 0.9736) (Fig. 5a). This result suggests an excellent concordance between our current dosimetry method and PLANET® Dose. According to the Bland–Altman plot method (Fig. 5b–d), the “bias” value (i.e. the mean of the AD differences between PLANET® Dose and Dosimetry Toolkit + OLINDA) was − 0.13 Gy for liver, 0.22 Gy for kidneys, and 0.30 Gy for spleen. Moreover, this approach estimated that the difference of AD values between methods ranged from − 0.75 to 0.49 Gy, from − 0.20 to 0.64 Gy, and from − 0.43 to 1.03 Gy for 95% of the 40 liver, kidneys and spleen dosimetry analyses, respectively. For liver, the Bland–Altman analysis showed that the mean absolute dose differences progressively increased with the dose (*R*^2^ = 0.5742).

#### Calculation method assessment

The comparison of the mean AD obtained with PLANET® Dose LDM and DK and using the TIACs from PLANET® Dose uploaded on OLINDA/EXM® V1.0 (i.e. the reference in this comparison) (Additional file 2: Fig. S1 and Table 6) showed that the mean relative difference and RMSD values were lower than 5% for all organs, but for spleen (maximum RMSD value of 6.5%).

**Table 6:** Mean, SD, range, RMSD value of the AD values to liver, kidneys and spleen calculated with PLANET® Dose + OLINDA and PLANET® Dose with density correction

	Absorbed dose (Gy)														
	Liver					Kidneys					Spleen				
	PLANET + OLINDA/EXM® V1.0	PLANET®Dose LDM	PLANET®Dose DK	Relative deviation (%)/PLANET + OLINDA		PLANET + OLINDA/EXM® V1.0	PLANET®Dose LDM	PLANET®Dose DK	Relative deviation (%)/PLANET + OLINDA		PLANET + OLINDA/EXM® V1.0	PLANET®Dose LDM	PLANET®Dose DK	Relative deviation (%)/PLANET + OLINDA	
				PLANET®Dose LDM	PLANET®Dose DK				PLANET®Dose LDM	PLANET®Dose DK				PLANET®Dose LDM	PLANET®Dose DK
Mean	3.43	3.27	3.20	− 4.5	− 6.2	3.31	3.25	3.17	− 1.9	− 4.2	4.47	4.45	4.33	− 1.1	− 3.4
SD	3.84	3.65	3.58	3.6	3.6	3.58	3.58	3.58	3.6	3.6	3.58	3.58	3.58	3.6	3.6
Min	0.62	0.61	0.60	− 6.6	− 8.5	1.97	1.94	1.91	− 5.8	− 7.7	2.40	2.36	2.31	− 5.5	− 7.6
Max	16.40	15.89	15.56	0.9	− 1.0	7.36	7.09	6.93	2.9	− 0.6	7.58	7.30	7.13	6.5	4.2
RMSD				4.9	6.5				2.5	4.4	4.6			3.3	4.6

The estimated Lin’s concordance correlation coefficient was 1 (*R*^2^ = 0.9966) (Additional file 3: Fig. S2a). The “bias” value of the Bland–Altman plot analysis (i.e. the average of the differences between PLANET® Dose LDM and PLANET® Dose + OLINDA) was − 0.16 Gy for liver, − 0.06 Gy for kidneys, and − 0.04 Gy for spleen. The difference of AD values between methods ranged from − 0.57 to 0.24 Gy, from − 0.18 to 0.06 Gy, and from − 0.34 to 0.26 Gy for 95% of the 40 liver, kidneys and spleen dosimetry analyses, respectively (Additional file 3: Fig. S2b–d). For liver, the negative trend (Bland–Altman analysis) showed an *R*^2^ value of 0.8358.

## Discussion

Due to the need of implementing central dosimetric data processing for multicentre trials, our department acquired a vendor-neutral solution, PLANET® Dose, for dosimetry assessments. However, to take advantage of the experience and data already acquired with Dosimetry Toolkit® and OLINDA/EXM® V1.0, we needed to compare their results. Overall, the two systems gave similar dosimetry results in the phantom study (CF and TIAC) and also when using imaging data from patients who received two injections of [^177^Lu]Lu-DOTA-TATE (mean organ masses, TIACs and ADs for liver, kidneys and spleen).

In a recent study, Huizing et al*.* [16] compared dosimetry results obtained with Hybrid Viewer Dosimetry and PLANET® Dose in ten patients using hybrid imaging data obtained from 0.5 h to 72 h post-injection of [^177^Lu]Lu-DOTA-TATE. Although our study also concerned PLANET® Dose, our reference method was Dosimetry Toolkit®. Furthermore, our study was based on complete 3D imaging data from 4 to 192 h post-injection in 21 patients undergoing [^177^Lu]Lu-DOTA-TATE treatment, thus representing a total of 40 dosimetry analysis.

In our study, the reconstruction step was performed with the GE application “Preparation for Dosimetry Toolkit” because PLANET® Dose does not include this functionality. The latter accepts reconstructed data supplied by others workstations, unlike the “Dosimetry Toolkit” application.

In a clinical dosimetry study, the preliminary step of calibration is crucial to obtain accurate activity quantification [28, 29]. A detailed calibration protocol is the cornerstone to achieve accurate and reliable image quantification in multicentric studies [30–33]. According to the GE recommendations, CF determined by planar acquisition of a 15 cm diameter Petri dish partially filled with a solution of ^177^Lu is sufficient for Dosimetry Toolkit®. However as our first aim was to monitor the AD to OARs, we used a large phantom (i.e. a bottle with a volume relatively close to that of kidney). The same methodology based on SPECT/CT imaging was followed for both calibration and clinical imaging. As described by Gustafsson et al*.* [34], SPECT image segmentation is essential to determine the activity concentration. For our phantom-based study, we selected a fixed volume threshold method based on an automatically drawn isocontour around the bottle with a volume of 200 mL. This volume was segmented on the SPECT images acquired at the first time point and rigidly copied to the others (i.e. rigid propagation). This step is directly affected by the accuracy of the registration of all SPECT/CT images using a rigid algorithm [11, 35, 36]. The obtained CF was similar (within 10%) to the one determined by Peters et al. [31] using a comparable gamma camera model.

Moreover, CFs are expressed in counts.s^−1^.MBq^−1^ by Dosimetry Toolkit® and in Bq.count^−1^ by PLANET® Dose. This implies that the CF must be modified with the acquisition duration for PLANET® Dose. This also highlights a risk of errors because of the lack of a standardized method for introducing CFs in dosimetry software tools. The crucial recommendation at this step is that the conditions used in clinical studies in terms of acquisition and reconstruction parameters must be similar to those used for calibration. We observed a negligible variation of the CF values over time, from *T* = 0–216 h. This means that the same CF can be used for each time point. This observation is particularly interesting for Dosimetry Toolkit® in which a single CF must be entered, whereas a different CF can be used at each time point with PLANET® Dose.

In the patient study, the liver masses obtained with the two packages were more similar than those obtained for smaller organs, such as kidneys and spleen, and the variability increased with the OAR decreasing size (Table 4b). Actually, as the DICOM-RT-Structure import could not be used in Dosimetry Toolkit®, organ delineation was done manually for each dosimetry package, leading to operator-induced variability. Thus, a small difference between contours could generate a more important relative deviation in smaller than larger organs. Moreover, the results presented in Table 5a–c seem to indicate a volume/mass effect. The RMSD of the AD values increased with the OAR decreasing size. For spleen, this could be explained by our package comparison methodology that used similar parameters in terms of registration and segmentation with constant volumes over time. Indeed, when using Dosimetry Toolkit®, the volume delineated on the first image was maintained, but adjusted by translation or rotation at each successive time point, when necessary. However, this step was not available in PLANET® Dose. Therefore, to use similar approaches with both packages, in PLANET® Dose we chose to perform an organ-based registration followed by rigid propagation (i.e. the contour registered in the first CT image was exactly propagated to the images acquired at the other time points). Thus, for some patients, the initial spleen contour did not fully match the organ contour at the other time points, and included tissues with different density. This implied an important deviation when calculating the AD with density correction. For instance, in the dosimetry analysis n°31, part of the spleen contour was moved to the left lung at other time points, and the AD was overestimated because of the density correction. For kidneys, AD variations between packages could be explained by the fact that in PLANET® Dose, left and right kidney are considered separately, while in OLINDA/EXM, the two kidneys are considered as a single organ. This may affect pharmacokinetic assessments, especially when rigid registration is considered. Therefore, we tested an additional approach that uses the segmentations and TIACs provided by PLANET® Dose and the dosimetric results (ADs) obtained with PLANET® Dose LDM or DK and with OLINDA/EXM® V1.0 (Table 6). The differences between approaches were reduced when the comparison considered only the AD calculation. This is in agreement with the idea that registration and segmentation are major steps, and probably induce more variability then the AD calculation step. Grassi et al. [36] showed that ADs to organs are significantly affected by the registration algorithm used. Indeed, due to respiratory motion during SPECT/CT and organ deformation, registration errors can happen. Although this may not influence the results of our comparison, it is clear that additional studies on registration and time activity curve fitting are needed, by taking advantage of the additional possibilities available in PLANET® Dose. Moreover, for accurate registration, position reproducibility during the four SPECT/CT acquisitions is crucial. Thus, patient set-up and immobilization devices are strongly recommended.

The Bland–Altman plots showed that the biases were quite low for all organs. The limits of agreement were rather tight with a maximum value of approximately 1 Gy for spleen. Moreover, the results for liver highlighted a trend for slightly higher AD values obtained with Dosimetry Toolkit + OLINDA than PLANET® Dose when the AD increased. This trend was more pronounced for patients with liver metastases, i.e. high activity gradients within the liver. One hypothesis is that cross-absorbed doses (from photons) may be different for heterogeneous vs. homogeneous activity distributions. Another point to consider is the density (homogeneous for OLINDA vs. voxel-based and coming from the CT images for PLANET® Dose). This may influence the AD calculation, but it is not clear to which extent. This certainly deserves to be thoroughly investigated, probably using Monte Carlo modelling to take into account also the possible impact of local density corrections [37].

The concordance evaluation (Lin’s coefficient value of 0.99) highlighted an excellent agreement between methods. Moreover, the dosimetry results obtained using PLANET® Dose (AD to liver, kidneys and spleen of 0.45 ± 0.50 Gy/GBq, 0.45 ± 0.13 Gy/GBq and 0.62 ± 0.17 Gy/GBq respectively) are in agreement with those of the literature [38].

As proposed by Gear et al*.* [39] in a practical guidance paper, the uncertainties at each step of the dosimetry analysis should be determined to express the accuracy of the dosimetry results. Currently, PLANET® Dose allows evaluating the relative proportion of interpolation (between time points) and extrapolation (after the last time point). Regardless of the goodness of fit, this is a good indication of the relevance of time sampling, but it is not sufficient to fully characterize the uncertainty associated with the dosimetric workflow. Such study requires important developments and will be implemented in the future.

From a qualitative point of view, PLANET® Dose is a user-friendly commercial solution that proposes a wide range of tools for segmentation, and several analytic fit functions. The time necessary for a dosimetry analysis is significantly reduced. Therefore, considering the good agreement with our reference dosimetry method, the concordance of the dosimetry results with the literature, the added value of this software (easy contouring, wide choice of time activity curve fitting models, time saving), and the fact that the observed differences were explainable and clinically acceptable, we think that the PLANET® Dose software can replace our current dosimetry package without any correction for dosimetry analysis. We can now start to investigate the different methodological possibilities offered by PLANET® Dose, such as elastic registration and propagation, time integration activity with multiple exponentials, AD rate computation at the voxel level, DICOM-RT import and export of structures. Its potential can now be fully explored, particularly for the determination of the tumour AD and for investigating the AD-response correlations.

## Conclusion

In this work we compared the dosimetric results obtained with the software currently used in our department and with a new dosimetry software package available on the market. The ADs to OARs (liver, kidney and spleen) obtained with PLANET® Dose were concordant with those calculated with GE Dosimetry Toolkit® and OLINDA/EXM® V1.0, and in agreement with the literature. These results allow us to use PLANET® Dose in clinical routine for patient dosimetry after targeted radiotherapy with [^177^Lu]Lu-DOTA-TATE.

## Supplementary Information

**Additional file 1. Table S1:** Patients’ characteristics.

**Additional file 2. Fig. S1:** Box-and-whisker plots showing the ADs to liver (a), kidneys (b) and spleen (c) calculated using PLANET® Dose and PLANET® Dose+OLINDA, as well as the relative AD differences between PLANET® Dose and PLANET® Dose+OLINDA (d).

**Additional file 3. Fig. S2:** Dispersion around the 45° line of the AD pairs obtained with PLANET+OLINDA and PLANET® Dose LDM with density correction for all organs combined (a). Bland-Altman plots of the AD to liver (b), kidneys (c) and spleen (d) calculated with PLANET+OLINDA and PLANET® Dose LDM with density correction.

## Data Availability

Please contact the author for data requests.

## Abbreviations

AD: Absorbed dose
CF: Calibration factor
DICOM-RT: Digital imaging and communications in medicine-radiotherapy
DK: Dose kernel
DTK: Dosimetry toolkit®
FOV: Field of view
GE: General Electric
IDAC: Internal dose assessment by computer
LDM: Local deposition method
MIRD: Medical internal radiation dose
NETs: Neuroendocrine tumours
NM: Nuclear medicine
OAR: Organ at risk
OSEM: Ordered subset expectation maximization
PRRT: Peptide receptor radionuclide therapy
RMSD: Root mean square deviation
SD: Standard deviation
SPECT/CT: Single photon emission computed tomography/computed tomography
TACs: Time activity curves
TIA: Time integrated activity
TIAC: Time integrated activity coefficient
VOI: Volume of interest
