# Supplementary material for: Clinical implementation of PLANET® Dose for dosimetric assessment after [177Lu]Lu-DOTA-TATE: comparison with Dosimetry Toolkit® and OLINDA/EXM® V1.0
Source: EJNMMI Res. 2021 Jan 4;11:1. doi: 10.1186/s13550-020-00737-8 (PMC7782649; doi:10.1186/s13550-020-00737-8)
Supplement: Supplementary file 1 — Additional file 1. Table S1: Patients’ characteristics. [file 13550_2020_737_MOESM1_ESM.docx]

| Patient | Sex | Age (years) | Weight  (kg) | | Primary tumour | Metastases | Injected activity (MBq) | | Number of treatment cycles | SPECT/CT  acquisition times | |
| --- | --- | --- | --- | --- | --- | --- | --- | --- | --- | --- | --- |
|  |  |  |  |  |  |  | *C 1* *C* 2 | |  | ***C 1*** | ***C* 2** |
| 1 | M | 41 | 84 | Pancreas NET | | - | 7284 | 7213 | 4 | 4h, 24h, 72h, 192h | 4h, 24h, 72h, 192h |
| 2 | M | 74 | 70 | Small intestine NET | | Nodes, mesentery | 7288 | 7578 | 3 | 4h, 24h, 72h, 192h | 4h, 24h, 72h, 192h |
| 3 | M | 61 | 74 | Small intestine NET | | Nodes, mesentery, liver, bone | 7298 | 7210 | 4 | 4h, 24h, 72h, 192h | 4h, 24h, 72h, 192h |
| 4 | F | 82 | 57 | Pancreas NET | | Liver | 7177 | 7239 | 4 | 4h, 24h, 72h, 192h | 4h, 24h, 72h, 192h |
| 5 | M | 75 | 74 | Small intestine NET | | Nodes, mesentery, liver | 7167 | 7073 | 4 | NA | 4h, 24h, 72h, 192h |
| 6 | M | 59 | 72 | Small intestine NET | | Nodes, liver, bone | 7287 | † | 1 | 4h, 24h, 168h | † |
| 7 | M | 71 | 71 | Pancreas NET | | Liver | 7054 | 7134 | 4 | 4h, 24h, 72h, 192h | 4h, 24h, 192h |
| 8 | M | 53 | 70 | Small intestine NET | | Liver, bone | 7102 | 7260 | 3 | 4h, 24h, 72h, 192h | 4h, 24h, 72h, 192h |
| 9 | F | 73 | 70 | Small intestine NET | | Liver, peritoneum | 7559 | 7384 | 4 | 4h, 24h, 72h, 192h | 4h, 24h, 72h, 192h |
| 10 | F | 63 | 56 | Pancreas NET | | Liver | 7323 | 7071 | 4 | 4h, 24h, 72h, 192h | 4h, 24h, 72h |
| 11 | M | 82 | 70 | Small intestine NET | | Nodes, mesentery, bone | 7180 | 6642 | 2 | 4h, 24h, 72h | 4h, 24h, 72h, 192h |
| 12 | M | 59 | 79 | Small intestine NET | | Mesentery, liver | 7207 | 7188 | 4 | 4h, 24h, 72h, 192h | 4h, 24h, 72h, 192h |
| 13 | M | 70 | 88 | Small intestine NET | | Liver, bone | 7222 | 7158 | 4 | 4h, 24h, 192h | 4h, 24h, 72h, 192h |
| 14 | M | 78 | 89 | Large intestine NET | | Nodes, liver | 7162 | 6620 | 2 | 4h, 24h, 72h, 192h | 4h, 24h, 192h |
| 15 | F | 48 | 37 | Small intestine NET | | Nodes, liver | 6595 | 7322 | 4 | 4h, 24h, 192h | 4h, 24h, 72h, 192h |
| 16 | M | 56 | 73 | Small intestine NET | | Nodes | 7317 | 7359 | 4 | 4h, 24h, 72h, 192h | 4h, 24h, 72h, 192h |
| 17 | M | 59 | 86 | Small intestine NET | | Nodes, liver | 7321 | 7300 | 4 | 4h, 24h, 72h, 192h | 4h, 24h, 72h, 192h |
| 18 | M | 68 | 66 | Small intestine NET | | Liver | 7327 | 7425 | 4 | 4h, 24h, 192h | 4h, 24h, 192h |
| 19 | M | 71 | 94 | Small intestine NET | | Nodes, liver | 7500 | 7201 | 4 | 4h, 24h, 72h, 192h | 4h, 24h, 72h, 192h |
| 20 | F | 79 | 39 | Small intestine NET | | Nodes, liver | 7489 | 7235 | 4 | 4h, 24h, 192h | 4h, 24h, 72h, 192h |
| 21 | F | 53 | 163 | Small intestine NET | | Nodes, liver | 7459 | 7153 | 4 | 4h, 24h, 72h | 4h, 24h, 72h, 192h |
| NA: Not available for dosimetry analysis; C: cycle; NET: neuroendocrine tumor; †: Patient dead | | | | | | | | |  |  |  |

Suppl. Table 1**:**
