# Supplementary figures and images for: Clinical implementation of PLANET® Dose for dosimetric assessment after [177Lu]Lu-DOTA-TATE: comparison with Dosimetry Toolkit® and OLINDA/EXM® V1.0
Source: EJNMMI Res. 2021 Jan 4;11:1. doi: 10.1186/s13550-020-00737-8 (PMC7782649; doi:10.1186/s13550-020-00737-8)

Suppl. Fig. 1**:**

**
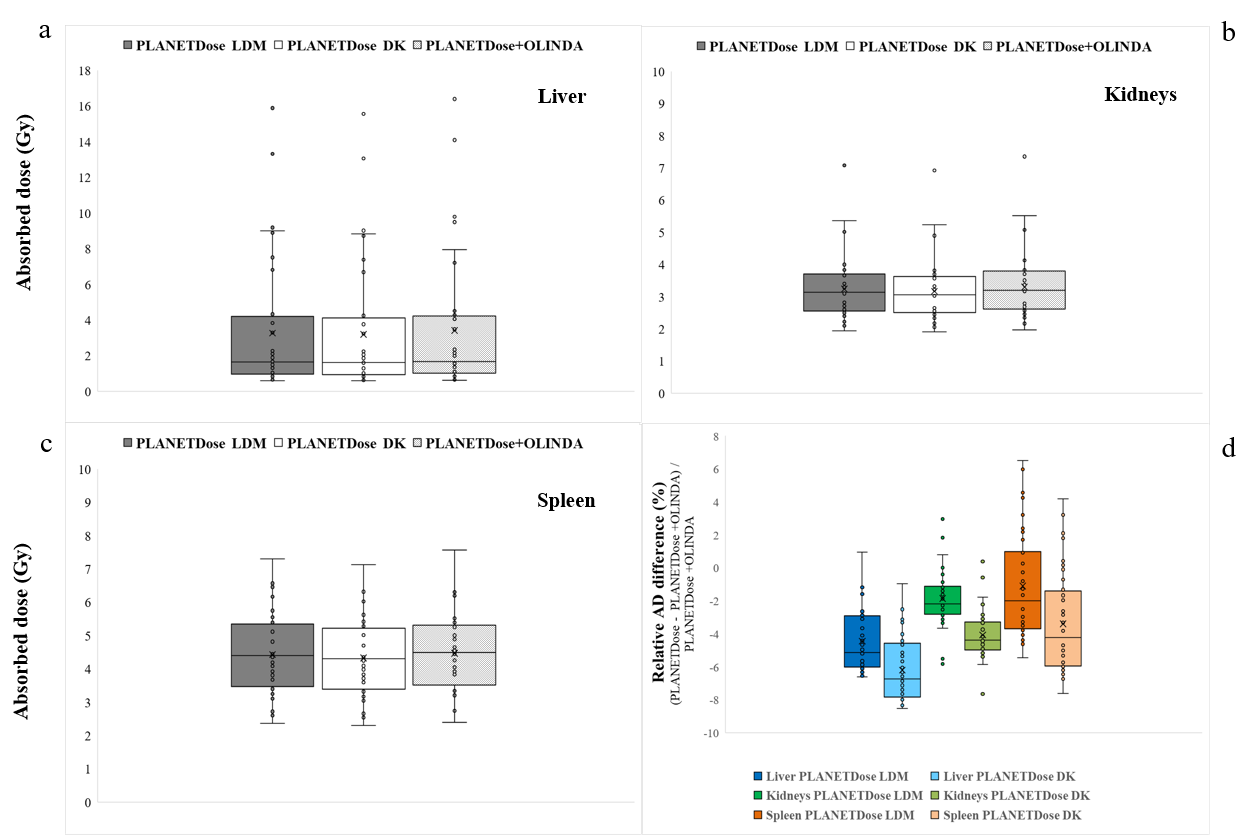
**

Supplement: Supplementary file 2 — Additional file 2. Fig. S1: Box-and-whisker plots showing the ADs to liver (a), kidneys (b) and spleen (c) calculated using PLANET® Dose and PLANET® Dose+OLINDA, as well as the relative AD differences between PLANET® Dose and PLANET® Dose+OLINDA (d). [file 13550_2020_737_MOESM2_ESM.docx]

Suppl. Fig. 2:


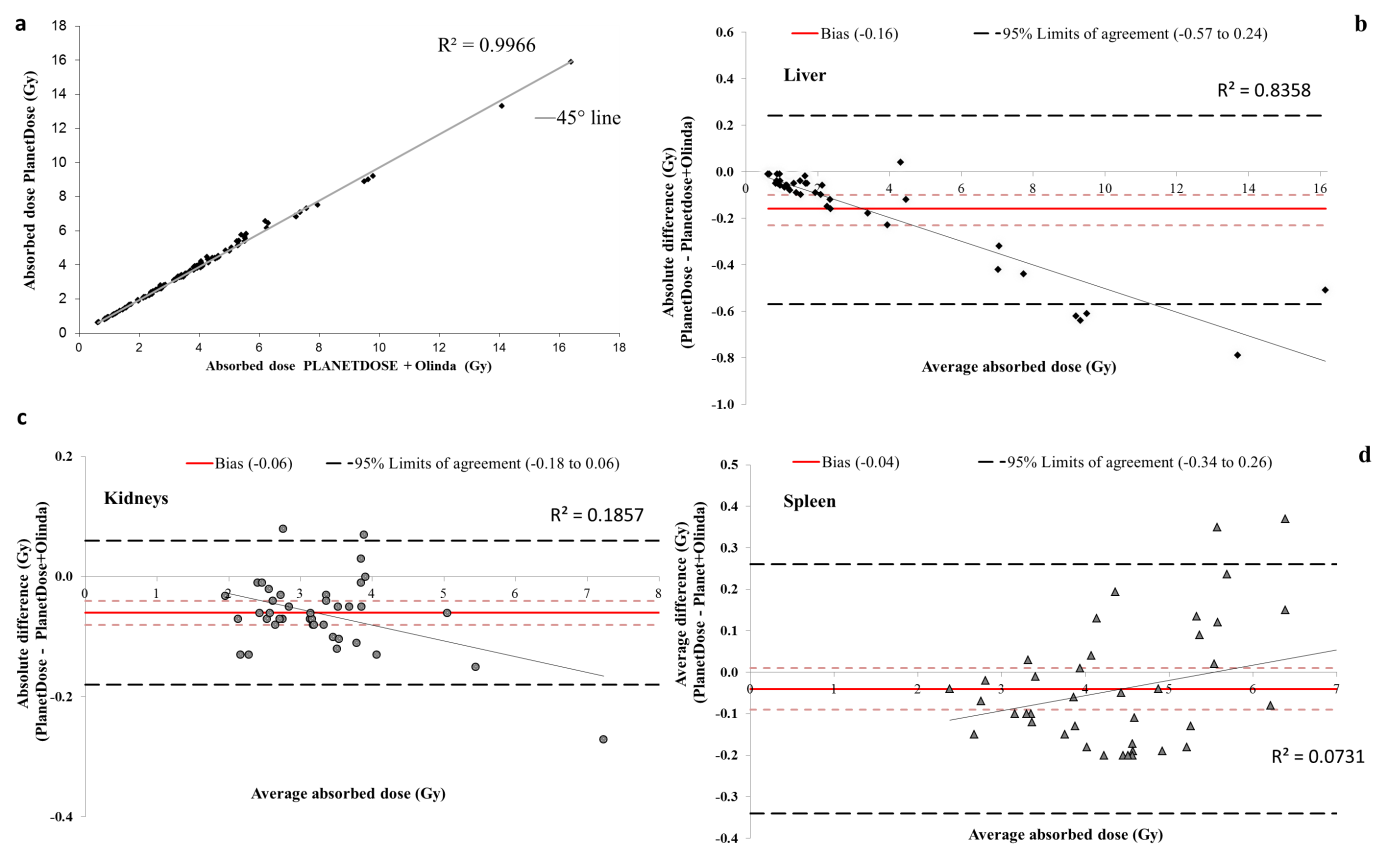

Supplement: Supplementary file 3 — Additional file 3. Fig. S2: Dispersion around the 45° line of the AD pairs obtained with PLANET+OLINDA and PLANET® Dose LDM with density correction for all organs combined (a). Bland-Altman plots of the AD to liver (b), kidneys (c) and spleen (d) calculated with PLANET+OLINDA and PLANET® Dose LDM with density correction. [file 13550_2020_737_MOESM3_ESM.docx]
